# The effect of dental rehabilitation under general anesthesia on dental anxiety in children: a systematic review and meta-analysis

**DOI:** 10.1186/s12903-025-07334-y

**Published:** 2025-12-29

**Authors:** Elham Keykha, Samira Hajisadeghi, Mohammad Taha Heidari

**Affiliations:** 1 Research Center for Prevention of Oral and Dental Diseases, Baqiyatallah University of Medical Sciences, Tehran, Iran; 2 School of Dentistry, Baqiyatallah University of Medical Sciences, Tehran, Iran

**Keywords:** Dental fear, Dental anxiety, Dental general anesthesia, Dental rehabilitation under general anesthesia, Systematic review, Meta-analysis

## Abstract

**Background:**

Dental fear and anxiety (DFA) are common in children, which can hinder their access to dental care and lead to poor oral health outcomes. Dental general anesthesia (DGA) is frequently used for uncooperative children or those requiring extensive treatment, enabling efficient care in a single session. However, its psychological effects on DFA—whether it alleviates or worsens anxiety—remain unclear. This highlights the need for a systematic review and meta-analysis to evaluate DGA's impact on children's DFA.

**Methods:**

A systematic search was conducted in PubMed, Scopus, Web of Science, and Google Scholar up to January 15, 2025, to identify observational and clinical studies on DFA in children (ASA I–II) under 12 years old before and after DGA or compared to those without DGA. Risk of bias was assessed by the NIH quality assessment tool for before-and-after studies without an external control, and the Newcastle–Ottawa scale for case–control and cohort studies. Results were synthesized using standardized mean differences (SMD) and 95% confidence intervals (CI), with evaluations for heterogeneity and publication bias.

**Results:**

Twelve studies were included in the review, with nine eligible for meta-analysis. Based on the NIH quality assessment tool, four studies were rated as good and three as fair. Three studies received medium quality scores on the Newcastle–Ottawa scale. The analysis revealed no statistically significant changes in DFA after DGA (SMD = -0.7, 95% CI: -1.993 to 0.593, *p* = 0.289), nor between DGA and non-DGA groups (CFSS-DS: SMD = 0.132; MCDASf: SMD = 0.850). High heterogeneity and possible publication bias were observed. The certainty of evidence was rated as very low by GRADE.

**Conclusion:**

There was no statistically significant evidence that DGA increases or decreases DFA in children. Due to the variability in outcomes and assessment methods, future research should use standardized tools, consider confounding factors, and include longer follow-up periods to better understand the psychological effects of DGA.

**Supplementary Information:**

The online version contains supplementary material available at 10.1186/s12903-025-07334-y.

## Introduction

Dental fear (DF) refers to a negative reaction to specific stimuli associated with dental treatment, while dental anxiety (DA) is characterized by an excessive or debilitating emotional response experienced by patients in a dental setting. Both terms are indicators of the early signs of dental phobia (DP), which is an irrational or extreme fear and anxiety related to dental examinations and treatments [1, 2]. Dental fear and anxiety (DFA) often result from negative childhood dental experiences but can develop later in life. Research has produced inconsistent findings regarding the links between DFA and sociodemographic factors, including age, gender, education, race/ethnicity, socioeconomic status, geographic location, and culture [3–5]. This condition can significantly affect daily life and lead to the prolonged avoidance of dental care. Abnormal DFA experienced during childhood or adolescence can often be associated with negative behaviors and outcomes related to oral health [5, 6]. Additionally, dentists often find anxious patients more challenging to manage and require more time to treat. Moreover, anxious patients frequently postpone their dental treatments and miss appointments, which can lead to more complicated treatment needs [7]. In many countries, DFA in children has been recognized as a public health concern [8].

To help reduce anxiety in children and enhance their cooperation during dental treatment, both pharmacological and non-pharmacological behavior guidance techniques (collectively called Dental Behavioral Support, or DBS) are recommended [9–12]. Most children can be effectively treated using local anesthesia; however, general anesthesia (GA) may be necessary for those who show poor compliance, are very young, or have significant medical conditions, disabilities, or extensive treatment needs, such as severe early childhood caries (ECC) [13]. GA is a pharmacological option that allows dental practitioners to complete all necessary dental procedures in one visit [12]. It is usually preferable to avoid multiple sedation visits for uncooperative children with extensive tooth decay, as repeated stressful procedures can decrease patient cooperation. Treatment under GA can be an effective solution for children who need urgent, comprehensive oral health care but are uncooperative [14, 15]. Unfortunately, dental treatment under general anesthesia (dental general anesthesia or DGA) can be an emotional and traumatic experience for children because of the stress of anesthesia induction, separation anxiety, and possible complications [16]. Since DFA itself is a leading reason for GA referrals [16], there is concern that GA might not address the underlying psychological distress and could even reinforce a negative feedback loop of fear and avoidance. If the GA experience contributes to future dental anxiety, its use as a routine management tool requires closer examination. Therefore, exploring the emotional and behavioral outcomes of DGA is crucial for helping clinicians choose treatment strategies that enable immediate care and encourage long-term patient cooperation.

Multiple studies have explored the relationship between DGA experience and its impact on DFA. Findings on how DGA affects DFA are mixed. A study found that DGA offers a painless treatment option that can help lower DFA [17]. Some studies reported a decrease in children's DFA levels following DGA [15, 18]. However, some research indicates that DFA is associated with previous DGA experiences, suggesting that children who have undergone DGA often exhibit higher anxiety levels than children who have not had a DGA history [12, 19]. Additionally, when researchers assessed changes in DFA after DGA compared to before the procedure, they observed an increase in DFA within weeks after surgery [20–22]. Conversely, some studies reported either no significant change in children's DFA levels following DGA [23–25]. Given the conflicting evidence, a systematic review is needed to assess whether DGA reduces or worsens DFA in children. This review will analyze data to clarify DGA's effects on DFA, guiding pediatric dentists, anesthesiologists, and policymakers to improve care strategies.

## Materials and methods

### Study design

This research is a systematic review and meta-analysis following the Preferred Reporting Items for Systematic Reviews and Meta-Analyses (PRISMA) guidelines [26].

### Protocol and registration

The protocol for this study has been registered with the International Prospective Register of Systematic Reviews (PROSPERO) under the ID CRD42025639768.

### Search strategy

A comprehensive search of the PubMed, Web of Science, and Scopus databases was conducted using a defined strategy and targeted keywords to identify relevant studies up to January 15, 2025. In addition to database searches, we looked for gray literature using Google Scholar (scholar.google.com) on that day. We did not set any date restrictions. Results were sorted by relevance, and the first 10 pages (~ 100 records) were exported and screened because our protocol specified screening of the top-ranked results. Furthermore, MTH, a co-author of this manuscript, manually examined the reference lists of all included studies and relevant reviews to identify any additional records not found through the electronic search. The keywords used in these searches included dental anxiety, dental fear, dental phobia, dental stress analysis, general anesthesia, and dental general anesthesia. In PubMed (MEDLINE), we combined MeSH headings with free-text terms using Boolean operators (OR within concept sets; AND across concepts; NOT to exclude irrelevant records). Core MeSH terms included “Dental Anxiety” OR “Dental Stress Analysis”, and “Anesthesia, General,” paired with synonyms as text words (e.g., "Dental fear" OR "Dental Phobia" OR "dental stress" OR "Odontophobia" AND "general anesthesia" OR "dental general anesthesia"). In Scopus and Web of Science (which do not use MeSH), we searched title/abstract/topic fields with equivalent Boolean strings. Full reproducible strategies are detailed in Supplementary Table 1. The search was independently carried out by two authors, EK and MTH.

### Eligibility criteria

The search strategy used the PICO method, which stands for Population, Intervention, Comparison, and Outcome. The main research question of this review was: "Does DGA affect DFA levels in healthy children?" The components of the PICO framework were defined as follows: Population (P): Healthy children (classified as American Society of Anesthesiologists physical status I and II, ASA I and II) under 12 years old receiving dental care; Intervention (I): Dental treatment performed under general anesthesia (DGA); Comparison (C): Pre-treatment DFA levels in the same children or matched children who did not undergo DGA; Outcome (O): Change in DFA levels, measured using validated psychometric tools.

We included studies with observational, cross-sectional, case–control, clinical trial, and cohort designs. Only studies published in English in peer-reviewed journals or presented at conferences were considered. The studies examined the mean and standard deviation of DFA levels in healthy children (ASA I and II) before and after they underwent DGA. Additionally, some studies compared these DFA levels to those of children who had not experienced DGA. All studies used validated and reliable psychometric questionnaires to assess dental anxiety, either through self-assessment or evaluations by caretakers. We excluded in vitro and animal studies unrelated to the research, articles lacking relevant or accurate data, and those without accessible full texts in English.

### Study selection

The articles retrieved during the search were first entered into EndNote software, where duplicates were identified and removed. Two authors, EK and MTH, independently screened the titles and abstracts of the studies, and any disagreements were resolved through discussion. When consensus was not achieved, EK, the pre-specified adjudicator, made the final decision based on their senior expertise and operational feasibility; disagreements were infrequent. No automation tools were used in the selection or data collection. Following the Cochrane protocol, the next step involved screening the titles and abstracts of the articles. Unrelated articles were removed at this stage, and the full texts of relevant or questionable articles were then evaluated. Finally, the data from the articles meeting the inclusion criteria for the meta-analysis were extracted.

### Data collection process and data items

We used a structured Excel data-extraction sheet that was iteratively refined during early extractions; although it was not formally pilot-tested, item definitions were harmonized before full extraction. Two reviewers (EK and MTH) independently extracted data, and disagreements were resolved through discussion. Authors of articles lacking full texts or facing issues such as data discrepancies, missing standard deviations, or inconsistencies were contacted. We extracted key information from the eligible articles, including the first author’s name, publication year, country of study, study design, number of participants, participant ages, the method used to measure DFA levels as assessment tools (e.g., Children’s Fear Survey Schedule-Dental Subscale (CFSS-DS), Modified Child Dental Anxiety Scale (MCDAS), Modified Child Dental Anxiety Scale (MDAS)), follow-up duration, quality score of each article, and main findings. This information included the mean and standard deviation (SD) of DFA levels before and after DGA, as well as a comparison of DFA levels between those who experienced DGA and those who did not.

### Risk of bias in individual studies

We selected risk-of-bias tools based on the study design. For pre–post studies without an external control group, we used the National Institutes of Health (NIH) quality assessment tool because it is specifically designed for these designs and emphasizes key threats to internal validity, such as prespecified outcomes, follow-up, outcome measurement, and attrition [27]. This tool includes a checklist that assesses the quality of studies based on 12 specific items. Each item is rated as "yes," "no," or "cannot be determined (CD)/not reported (NR)/not applicable (NA)." At the end of the assessment, articles are classified as good, fair, or poor according to their scores: less than 5 is poor, 5–8 is fair, and above 8 is good. The 12 checklist items cover aspects such as study question, eligibility criteria, study population, study participants, eligible participants, sample size, intervention, outcome, blinding, follow-up rate, statistical analysis, multiple outcome measures, group-level interventions, and individual-level outcome efforts. For cohort and case–control studies, we employed the Newcastle–Ottawa Scale (NOS), which is widely used for these designs and measures bias across selection, comparability, and outcome/exposure domains, allowing transparent, design-specific judgments [28]. The checklist scores range from zero to nine. We classified the articles into three quality levels: low quality (scores less than 3), medium quality (scores between 3 and 6), and high quality (scores above 6). These selections align the appraisal method with the foundational study designs and current practices in the field, thereby improving comparability with existing research. Primary risk-of-bias assessments were conducted by MTH using a standardized template, under the pre-specified supervision of EK (senior methodological/subject-matter expertise). Supervision included calibration, written decision rules, and review of ambiguous cases [29]; disagreements were discussed and, if unresolved, were settled by EK.

### Summary measures and synthesis of results

Only studies reporting the mean and standard deviation of DFA scores were included in the meta-analysis, focusing on those using the same assessment tools or follow-up durations. Studies lacking sufficient quantitative data were described narratively but excluded from the pooled analysis. When necessary data was missing or unclear, the corresponding authors were contacted for clarification. No imputation was performed for missing outcome data. The results from individual studies were summarized in structured tables and forest plots, showing effect estimates and confidence intervals for all outcomes included.

The extracted data were initially entered into Excel for adjustments and finalization, after which all statistical analyses were performed using Stata software version 16 (Stata Corp, College Station, TX, USA).

We used the Chi-square test and the I-square statistic to evaluate heterogeneity among the included studies. An I-square value above 50% was considered indicative of substantial heterogeneity. Based on the heterogeneity analysis results, we applied the random-effects model when heterogeneity was significant and the fixed-effect model when heterogeneity was low to pool the results. Potential publication bias was evaluated through visual inspection of funnel plots, as Egger’s test is not recommended with fewer than 10 studies. Because this meta-analysis involved comparisons of means, the results are shown as the standardized mean difference (SMD) with a 95% confidence interval (95% CI), serving as the summary measures. No subgroup analyses or meta-regressions were performed due to the small number of studies and the variability in outcome measures and follow-up periods. Sensitivity analysis was conducted by sequentially removing individual studies to evaluate their impact on the overall pooled effect size.

The certainty of evidence was evaluated using the GRADE framework, taking into account risk of bias, inconsistency, indirectness, imprecision, and publication bias [30, 31]. Since all included studies were observational, the initial certainty was rated as low and was further downgraded if significant issues arose in any of these areas [32].

## Results

### Study selection

**Fig. 1:**
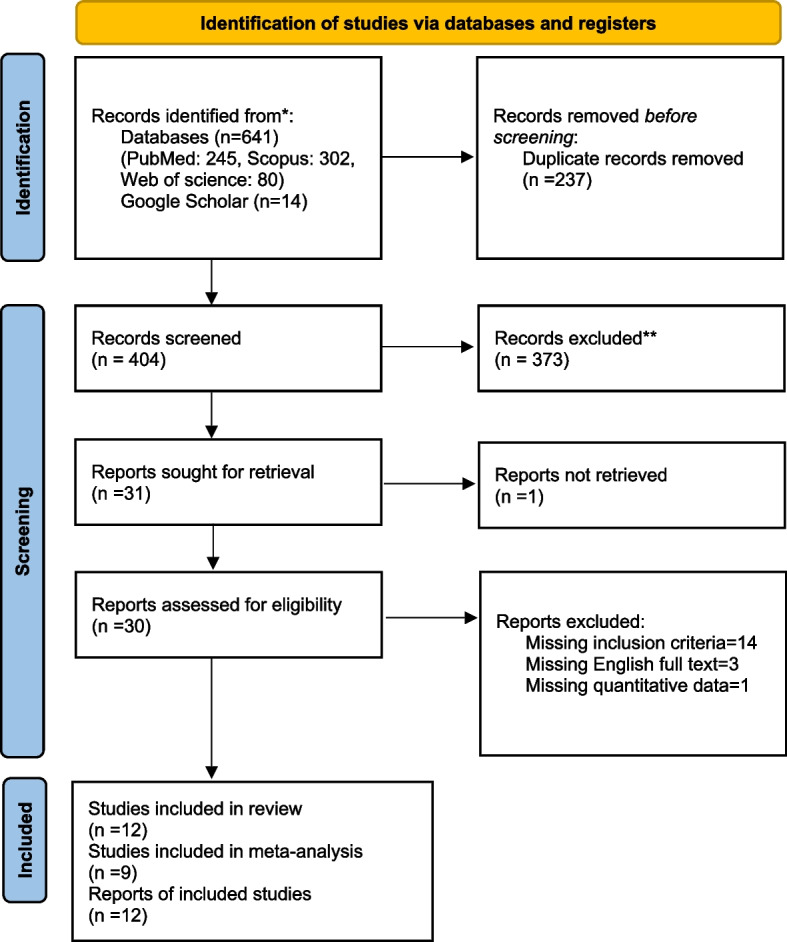
Flow diagram of the literature search for studies included in the meta-analysis

Figure 1 presents the steps taken to identify studies, eliminate duplicates, and screen them based on their titles, abstracts, and full texts. After conducting searches in databases, we identified a total of 641 studies. Upon removing duplicates, 404 studies remained for screening. Two authors independently reviewed the titles and abstracts, excluding 374 studies that did not meet the inclusion criteria. The full texts of the remaining 30 studies were then analyzed. Ultimately, 12 studies remained and were summarized in Table 1, and we could use 9 for meta-analysis [12, 19, 20, 25, 33, 35–38]. List of excluded articles with reasons (*n =* 18) is in Supplementary Table 2. The studies that were excluded primarily lacked the necessary data and English full text, used inappropriate DFA examination methods or timelines, and had comparison groups that did not fit our desired criteria or did not align with our study objectives or meet other inclusion requirements.

**Table 1:** Summary of the characteristics of the studies included in the systematic review

ID	Authors	Country	Year	Study design	Age (years)	Comparison groups	Time of DFA assessment	DFA scale	Relative results
1	Aldossari, G. S [19]	Saudi Arabia	2019	Cross-sectional	5–12 mea*n =* 9.15	DGA Cases vs control	History of DGA at least in one year ago	CFSS-DS MCDASf	Children who experienced DGA are a high-risk group for DFA several years after the procedure
2	Arch, L. M [23]	United Kingdom	2001	Cross-sectional	8–15 mea*n =* 11	Before DGA vs after DGA	Before DGA and one week post-treatment	MCDAS	The mean level of DFA did not reduce at post-treatment in the GA group
3	Cantekin, K [20]	Turkey	2014	Cross-sectional	4–7 mea*n =* 5.03	Before DGA vs after DGA	Before DGA and one to three weeks post-treatment	CFSS-DS	Children’s DFA increased
4	Duruk, G [33]	Turkey	2021	Cross-sectional	3–12 mea*n =* 5.90	Before DGA vs after DGA	Before DGA and one month post-treatment	CFSS-DS	The decreases in DFA in children were observed after DGA
5	Gazal G [34]	United Kingdom	2004	RCT	2–12 mea*n =* 5.9	Before DGA vs after DGA	Before DGA and on recovery from DGA, and again, 15-min following recovery	Five-point face scale	Extraction of teeth under GA does cause distress in children
6	Guney, S [35]	Turkey	2018	Cross-sectional	3–5 6–12 mea*n =* 5	Before DGA vs after DGA	Before DGA and one month post-treatment	Venham Picture Test(VPT) (3-5y) CFSS-DS (6-12y)	DFA decreased in 3–5 and 6–12 years old after DGA
7	Heaton, L [36]	United states of America	2023	Cohort	4–12 mea*n =* 5.8	Before DGA vs after DGA	Before DGA and 16 weeks post-treatment	CFSS-DS	DFA increased slightly, but not significantly
8	Klaassen, M [37]	Netherlands	2008	Cross-sectional	Under 8 mea*n =* 4.06	Before DGA vs after DGA	Before DGA and two weeks post-treatment	CFSS-DS	DFA did not change
9	Klaassen, M [25]	Netherlands	2009	RCT	Under 7 mea*n =* 4.08	DGA Cases vs control And Before DGA vs after DGA	Before DGA and one month post-treatment	CFSS-DS	In the total CFSS-DS scores no effects were found
10	Mathew, M [38]	Saudi Arabia	2023	cohort	Under 6 mea*n =* 4.21	Before DGA vs after DGA	Before DGA and 14 days post-treatment	CFSS-DS	Most participants were found to be less anxious and fearful toward dentists and dental treatment
11	Yıldırım, S [15]	Turkey	2018	Cross-sectional	3–6 mea*n =* 4.7	Before DGA vs after DGA	Before DGA and one month post-treatment	Facial Image Scale (FIS) Frankl's behavior rating scale (FBRS)	DGA can reduce the DFA level of children with a high amount of caries
12	Zhou, F [12]	China	2022	cross-sectional	4–6 mea*n =* 5.1	DGA Cases vs control	History of DGA within 2 years ago	MCDASf	The DFA score of the GA group was significantly higher than that of the control group

### Study characteristics

Table 2 summarizes the characteristics of the articles included in the meta-analysis. The earliest article was published in 2001, while the most recent appeared in 2023. Sample sizes ranged from 20 to 200 children. Seven studies measured DFA using the Dental Subscale of the Children's Fear Survey Schedule (CFSS-DS), developed by Cuthbert and Melamed in 1982. The CFSS-DS is a well-known, reliable tool with 15 items scored from 1 (not afraid) to 5 (very afraid), totaling 15–75. Scores above 38 indicate high dental fear [20, 39]. Two studies used the MCDAS, based on Corah’s scale, with 8 questions on dental procedures rated on a five-point scale from 5 (little or no anxiety) to 40 (extreme anxiety) [40, 41]. Most studies compared DFA scores before and after DGA, although some examined DFA after DGA compared to those without prior experience. The average age across studies was 5.4 years, and the median follow-up in before-and-after studies was 4 weeks (interquartile range 2–16 weeks). Two studies were cohort studies, one was a clinical trial, and six used a cross-sectional design.

**Table 2:** Summary of the characteristics of studies included in the meta-analysis

ID	Author	Year	Country	Study design	Age (year)	Pre GA DFA measurement	Pre GA cases (N)	Pre GA CFSS-DS mean	Pre GA CFSS-DS SD	Post GA DFA measurement	Post GA cases (N)	Post GA CFSS-DS mean	Post GA CFSS-DS SD	Risk score
1	Mathew, M [38]	2023	Saudi Arabia	Cohort	Under 6 mea*n =* 4.21	On the day of intervention	200	46.6	7.1	14 days	200	23.8	5.6	Good
2	Heaton, L [36]	2023	United states of America	Cohort	4–12 mea*n =* 5.8	At pretreatment	56	32.7	14.6	16 weeks	51	31.6	12.7	Good
3	Duruk, G [33]	2021	Turkey	Cross-sectional	3–12 mea*n =* 5.90	One week before the operation	43	38.93	14.03	1 month	43	25.67	15.81	Good
4	Guney, S [35]	2018	Turkey	Cross-sectional	6‐12	Before treatment	21	39.7	11.00	1 month	21	32.3	8.6	Fair
5	Cantekin, K [20]	2014	Turkey	Cross-sectional	4–7 mea*n =* 5.03	Before operation	311	32.7	9.3	1–3 weeks	311	37.8	11.2	Good
6	Klaassen, M [25]	2009	Netherlands	RCT	Under 7 mea*n =* 4.08	Before treatment	25	36.40	13.37	1 month	20	37.76	11.60	Fair
7	Klaassen, M [37]	2008	Netherlands	Cross-sectional	Under 8 mea*n =* 4.06	Before treatment	25	38.56	13.79	2 weeks	19	35.98	11.5	Fair
ID	Author	Year	Country	Study design	Age	DGA experience history	Control cases	Control CFSS-DS mean	Control CFSS-DS SD	DFA measurement time	GA cases	GA CFSS-DS mean	GA CFSS-DS SD	Risk score
1	Aldossari, G. S [19]	2019	Saudi Arabia	Cross-sectional	5–12 mea*n =* 9.15	At least one year ago	55	23.62	7.40	During study	43	34.33	10.44	Medium
2	Klaassen, M [25]	2009	Netherlands	RCT	Under 7 mea*n =* 4.08	During study procedure	29	38.47	16.55	2 weeks post DGA	14	31.12	11.88	Medium
3	Klaassen, M [25]	2009	Netherlands	RCT	Under 7 mea*n =* 4.08	During study procedure	23	42.6	13.43	2 weeks post DGA	20	37.76	11.60	Medium
ID	Author	Year	Country	Study design	Age	DGA experience history	Control cases	Control MCDASf mean	Control MCDASf SD	DFA measurement time	GA cases	GA MCDASf mean	GA MCDASf SD	Risk score
1	Zhou, F [12]	2022	China	Cross-sectional	4–6 mea*n =* 5.1	During 2 years ago	20	19.60	5.43	During study	20	21.65	7.49	Medium
2	Aldossari, G. S [19]	2019	Saudi Arabia	Cross-sectional	5–12 mea*n =* 9.15	At least one year ago	55	12.65	3.55	During study	43	20.40	7.78	Medium

### Risk of bias within studies

As shown in Table 2, we used the NIH tool to assess the risk of bias in before-and-after studies (seven studies). Four studies were rated as good quality, three as fair quality, and none as poor quality. The Supplementary Table 3 provides details on the evaluation criteria and quality scores.

We used the Newcastle–Ottawa assessment scale for case–control and cohort studies (three studies), with results presented as risk scores in Table 2. The complete Newcastle–Ottawa scale is available in the Supplementary Table 3. Overall, three studies received a medium-quality score, with none rated low or high.

### Comparison of CFSS-DS Pre-DGA vs Post-DGA in children

**Fig. 2:**
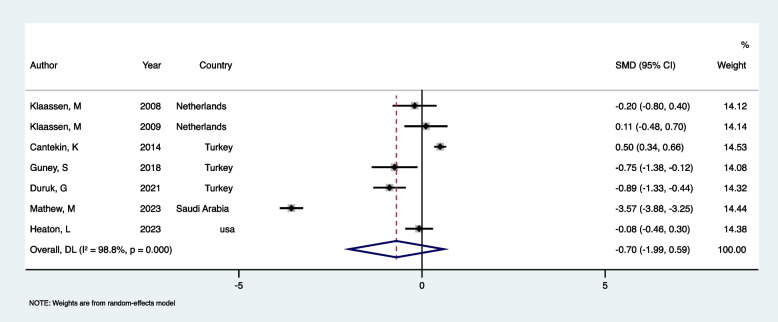
Forest plot comparing CFSS-DS Pre-DGA vs Post-DGA in children

In a review of seven studies [20, 25, 33, 35–38], DFA levels measured by CFSS-DS in healthy children (ASA I and II) were compared before and after they underwent DGA. A total of 673 children were included in the analysis, which revealed an SMD of −0.7 (95% CI: −1.993 to 0.593, *p* = 0.289) (Fig. 2, Table 3). The negative SMD suggests that DFA decreased after DGA; however, this decrease was not statistically significant, as the CI includes zero. This indicates there were no significant changes in DFA levels after DGA compared to pre-anesthesia levels. Additionally, the analysis of heterogeneity showed substantial variability between the studies (*I*^2^ = 98.8%, *p <* 0.001), resulting in the use of a random-effects model to synthesize the main results.

**Table 3:** Summary of meta-analysis results

Groups	Number of reports	Test of comparison				Heterogeneity		
		SMD (95% CI)	*P* value	Model	Z	Chi square	*P* value	I square (%)
CFSS-DS (Pre DGA vs Post DGA)	7	−0.7 (−1.993- 0.593)	0.289	Random	−1.061	517.64	< 0.001	98.8
CFSS-DS (DGA individuals vs control)	3	0.132 (−1.048 −1.313)	0.826	Random	0.220	26.95	< 0.001	92.6
MCDASf (DGA individuals vs control)	2	0.850 (−0.152- 1.853)	0.097	Random	1.662	6.90	0.009	85.5

**Fig. 3:**
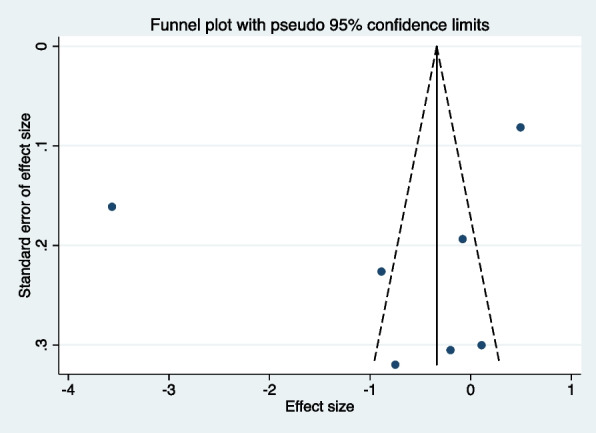
Funnel plot to check the publication bias

Sensitivity analysis was also performed, and the results showed that the most significant change occurred after removing Mathew, M. G., et al.’s study. The results remained consistent with previous analysis (SMD: −0.19, 95%CI: −0.71 to 0.32). Except for Heaton L et al.’s study, which measured DFA 16 weeks post-treatment, all other studies measured DFA up to 4 weeks. After excluding Heaton L et al.’s study, results showed no difference (SMD: −0.80, 95%CI: −2.36 to 0.76). In a leave-one-out (study-omission) sensitivity analysis, in all scenarios, 95% confidence intervals crossed 0, and overall conclusions did not change [42, 43]. Visual inspection of the funnel plot (Fig. 3) indicated potential publication bias due to asymmetry. Therefore, while bias cannot be completely ruled out, the findings should be interpreted with caution.

### Comparison of CFSS-DS in DGA individuals vs controls

**Fig. 4:**
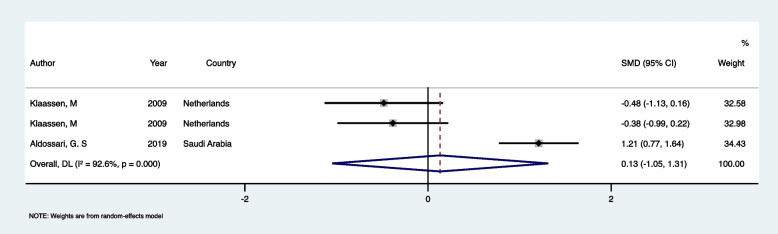
Forest plot comparing CFSS-DS in DGA individuals vs controls

**Fig. 5:**
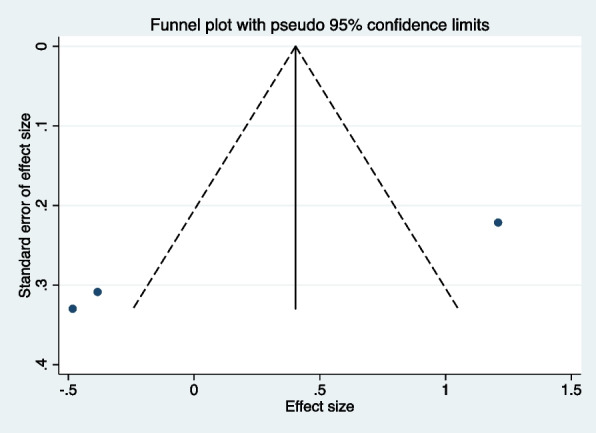
Funnel plot to check the publication bias

Only in two studies [19, 25] were DFA levels, as measured by CFSS-DS, compared between healthy children (ASA I and II) with experience of DGA and those without such experience. Klaassen et al. measured DFA in four groups, allowing comparison between paired groups. Ultimately, three data sets were included in the meta-analysis. The analysis found no significant difference in DFA levels between healthy children who underwent DGA and those who had not experienced DGA (SMD: 0.132, 95% CI: (−1.048 to 1.313), *p* = 0.826) (Fig. 4, Table 3). The heterogeneity among the studies was very high (*I*^2^ = 92.6%, *p <* 0.001), indicating substantial variation. Therefore, there is no clear evidence that DGA influences anxiety levels differently compared to the control group. The funnel plot (Fig. 5) suggests potential publication bias due to asymmetry. However, the Egger test result (t = −9.16, *p* = 0.069) does not provide significant evidence of bias.

### Comparison of modified child dental anxiety scale (MCDASf) in DGA individuals vs controls

**Fig. 6:**
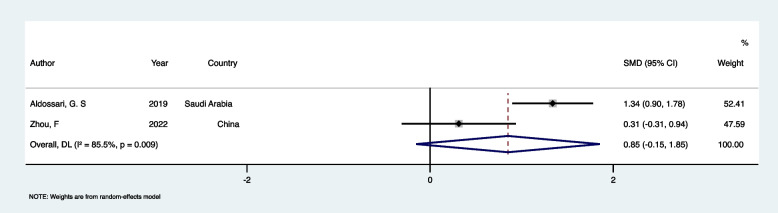
Forest plot comparing MCDASf in DGA individuals vs controls

In just two studies [12, 19], researchers compared dental anxiety levels, measured by the MCDASf, between healthy children (ASA I and II) who experienced DGA and those who did not. The analysis showed no significant differences in anxiety levels between the two groups (SMD: 0.850, 95% CI: (−0.152 to 1.853), *p* = 0.097) (Fig. 6, Table 3). However, the high heterogeneity among the studies (*I*^2^ = 85.5%, *p* = 0.009) indicates substantial variability in findings. Although there is a hint of a trend suggesting increased anxiety after DGA, the results are not statistically conclusive. Since only two studies were included in the meta-analysis, assessing publication bias using a funnel plot or Egger’s test was not possible.

### Certainty of evidence

Supplementary Table 4 presents the certainty of evidence assessment using the GRADE framework. For the primary outcome—the change in children’s DFA after DGA—the certainty of evidence was rated as very low. This rating reflects limitations in study design (observational before–and–after and cohort studies), very high heterogeneity (e.g., CFSS-DS pre/post: *I*^2^ = 98.8%), and imprecision (wide confidence intervals indicating possible benefits or harms). For the comparative analyses (DGA vs. control), the certainty of evidence was also very low due to high inconsistency (CFSS-DS: *I*^2^ = 92.6%; MCDASf: *I*^2^ = 85.5%), small sample sizes, and imprecision. Publication bias could not be reliably assessed when fewer than 10 studies contributed to a synthesis [44]; therefore, visual asymmetry should be interpreted cautiously, and Egger’s test was not performed in these cases.

## Discussion

### Interpretation

DFA is a global issue that affects people worldwide [45] and causes challenges for patients and dentists [46]. Childhood or adolescent dental fear can lead to harmful behaviors and poor oral health, driven by traumatic experiences, negative perceptions, or influence from family and peers. DFA is related to sociodemographic factors such as age, gender, education, ethnicity, socioeconomic status, location, and cultural background. Some avoid dental visits, resulting in untreated issues and poor hygiene. Studies show anxious patients have more decayed, missing teeth, and fewer fillings [47], which worsens oral health [4–7, 48]. The estimation of DFA can be influenced by the subjective nature of anxiety, which is often difficult to quantify [3]. Nevertheless, there are three primary methods used to measure anxiety: self-report questionnaires, physiological measures such as heart rate and perspiration, and observable behavioral indicators like avoiding eye contact and fidgeting. Questionnaires are particularly valuable for capturing subjective feelings of anxiety and pain [49–51]. Several validated tools are available to assess DFA in children and teens, including the Dental Fear Survey (DFS), Venham Picture Test (VPT), Children's Dental Fear Picture Test (CDFP), Children’s Fear Survey Schedule-Dental Subscale (CFSS-DS) and its faces version (CFSS-DSf), Modified Child Dental Anxiety Scale (MCDAS) and its faces version (MCDASf), Dental Anxiety Question (DAQ), and Facial Image Scale (FIS). Ideally, children self-report their anxiety, with adult assistance for younger children. Using reliable and valid tools suited to the age group is essential [3, 52, 53].

This study showed that DGA does not risk affecting DFA in children. The reduction in DFA levels after DGA, compared to pre-treatment levels, and the rise in DFA levels in children who experienced DGA versus those who did not were found to be statistically insignificant.

Based on our results, there was an insignificant decrease in DFA levels, as measured by the Children's Fear Survey Schedule-Dental Subscale (CFSS-DS), after DGA compared to pre-anesthesia levels. Most studies involved children with an average age of four to six years, except for Guney et al.’s study; excluding this study in the sensitivity analysis showed no difference (SMD: −0.70, 95% CI: −2.14 to 0.75). All studies measured DFA up to 4 weeks, except for Heaton L. et al.’s study, which assessed DFA 16 weeks post-treatment; after excluding this study as well, the results still showed no difference (SMD: −0.80, 95%CI: −2.36 to 0.76).

Some studies have reported results similar to ours [23–25, 35–37, 54]. These studies used CFSS-DS, MCDAS, and VPT assessment tools. They involved different age groups ranging from 3 to 15 years old, with an average age of 6.1 years. The follow-up periods varied from one week to 29 months. The studies employed various methodologies. Although these studies support our findings, some variables may have contributed to the lack of significant differences or heterogeneity in anxiety levels before and after DGA. For example, children referred for DGA often exhibit higher baseline DFA and poorer cooperation [19], and the most anxious individuals find the procedure more distressing [24]. These traits suggest a potential selection bias in the studies. This referral process can result in samples with highly anxious children, causing ceiling effects that restrict observed pre–post DFA changes and may even elevate post-operative anxiety, as highly anxious children perceive peri-operative events as threatening, thus biasing estimates toward the null or showing apparent DFA increases. Additionally, DFA scores are influenced by when and where questionnaires are administered and who completes them. Methodological guidance highlights that recall period and administration timing can systematically alter patient-reported outcome (PRO) scores, so these should be predefined and reported [55]. If anxiety forms are completed during multiple preoperative visits or later postoperative visits, familiarity and behavior guidance may have already reduced arousal, resulting in lower DFA scores [56]. Conversely, measures taken immediately before GA in a hospital setting tend to capture peak anticipatory distress in many children [57]. The informant also matters: child self-report, parent proxy, and clinician-rated measures can produce systematically different DFA scores. Agreement between child self-report and parent proxy is often moderate to poor, with parents frequently misestimating; thus, child self-report using validated scales is preferred when possible [58, 59]. Finally, elements of the clinical environment, such as clinician attire, can impact observed anxiety levels. A recent randomized trial indicated that child-friendly attire lowered anxiety during injections, although the overall evidence on “white-coat” effects varies depending on the setting [60, 61]. These factors contribute to the variability observed across studies, especially when questionnaires are administered at different times and locations by different informants.

Some studies showed results that were different from our meta-analysis findings [15, 18, 33, 35, 38, 62]. These studies used assessment tools like CFSS-DS, MCDASf, FIS, and VPT. They included children aged 3 to 12 years, with an average age of 4.8 years. The follow-up periods ranged from one week to two months. The observed reductions in anxiety may be due to the timing of postoperative DFA measurements and the different postoperative protocols used at various locations. Additionally, the connection between DFA and the positive effects of behavioral therapy suggests that ongoing communication from the dental team—from the first visit through postoperative check-ups after comprehensive DGA—helps lower children's DFA. Even if a child understands that their dental work is finished, knowing they only need to visit the dentist for follow-up appointments can also help reduce their anxiety [33, 38, 63, 64].

Some studies show children experience increased anxiety after DGA [20, 34, 65], using CFSS-DS and FIS tools. Participants aged 2–12, averaging 5.5 years, with follow-ups from one day to three weeks. Extracting teeth can distress children, and a higher number of extractions is linked to higher fear levels. Many felt unwell after the procedure, with some still experiencing symptoms a week. Anxiety was linked to distress during and after the procedure. Timing of measuring anxiety is crucial, with most studies having short-term follow-ups, explaining the increased DFA after DGA [20, 34, 65, 66].

In our meta-analysis, the SMD of the CFSS-DS and MCDASf scores in the DGA group was slightly but insignificantly higher compared to the control group. Only Klaassen et al. reported no differences in CFSS-DS scores between the GA group and the control group, nor significant changes in pre- and post-anesthesia DFA [25, 37]. It was a short-term study. In retrospective studies, experience with GA has been shown to contribute to DFA. Researchers Zhou, F. et al. and Aldossari, G. S. et al. compared children who had undergone DGA in at least one or two recent years with control groups. They concluded that children who experienced DGA are at a higher risk of developing DFA [12, 19]. Haworth, S. et al. found that participants aged 17 who underwent GA before the age of 7 exhibited notably higher DA than their peers who did not receive any GA [21]. It has also established a significant connection between DA in children and a history of prior GA [22]. However, in these studies, they did not measure anxiety before GA. On the other hand, as children mature, it is clear that their DFA significantly diminishes, which raises doubts about the accuracy of the results [67, 68].

Taken together, the findings indicate that DGA facilitates delivering comprehensive dental care but does not consistently reduce long-term DFA levels. Overall, the mixed evidence suggests that GA shouldn’t be seen as a standalone solution for managing anxious patients but rather as part of a broader spectrum of behavioral care.

### Limitations

A major limitation of this study is the heterogeneity of the collected data. This variability arises from several factors, including different methods of DFA measurement and the scales used, which can affect results and make data pooling in meta-analyses more difficult [3, 69, 70]. Other confounding factors may also contribute, such as age (since younger children generally have higher baseline anxiety) [67, 68]; gender (evidence is mixed on whether higher DFA levels in girls, while others see no difference or more anxiety in boys) [2, 71]; socioeconomic background and parental characteristics (as children's DFA is often linked to their parents' negative dental experiences and poor oral health) [36]; primary reasons for referring to DGA as child’s low cooperation and high anxiety, which may persist after anesthesia [19]; previous GA exposure (as prior experiences may increase anticipatory anxiety) [12, 19, 21, 22]; treatment burden (e.g., the number of tooth extractions, with more invasive procedures potentially increasing post-operative fear) [65]; as well as education, race/ethnicity, geographic location, and culture [3–5]. Additionally, the timing of anxiety measurements can introduce recall and maturation effects, and different postoperative DGA protocols across locations can also influence outcomes [38, 63, 64]. However, each study tracked variables such as participants' age ranges, gender, and history of systemic disease. While we cannot eliminate the possibility of publication bias, it is important to interpret the findings carefully [72]. Another limitation is that the certainty of evidence for all outcomes was rated very low by GRADE, mainly due to study design limitations, inconsistency, and imprecision [31, 32]. We recognize that not conducting an independent duplicate risk-of-bias assessment could introduce subjectivity. Although we use a standardized tool, criteria, calibration, and audits to lower this risk, methodological standards suggest that independent assessments are preferable to reduce errors and bias. This remains a limitation of our review [29, 73].

### Implications

Although evidence indicates that DGA does not affect DFA, clinicians should not choose DGA as the first-line approach for managing patients with DFA. DGA remains an option for children who cannot tolerate treatment with local anesthesia or conscious sedation, but behavioral guidance and parental coaching before and after GA are crucial to prevent a cycle of avoidance. Whenever possible, less invasive DBS techniques such as tell-show-do, desensitization, or inhalation sedation should be attempted first. Referral pathways must clearly differentiate between treatment facilitation (completing necessary dental care) and anxiety management.

It is recommended that future studies be designed to compare the level of DFA between children scheduled for DGA and those without such experience, serving as the control group. These studies should assess DFA both before and after treatment, with a longer follow-up period to evaluate possible changes over time. Additionally, all potential confounding factors, especially age and gender, should be analyzed to determine their impact on anxiety outcomes. A standardized anxiety measurement tool should be used at all study sites to ensure consistency and comparability of results. Furthermore, DGA should follow a unified clinical protocol, ideally across multiple countries, to reduce variability caused by procedural differences.

## Conclusion

This systematic review and meta-analysis found no statistically significant evidence that DGA increases or decreases DFA in children. Although DGA is often necessary for uncooperative children or those with extensive dental needs, its long-term psychological impact remains unclear. Our analysis suggests that while DGA does not significantly worsen DFA, it may also not reliably reduce it. Therefore, DGA should be carefully considered as part of a broader anxiety management approach, ideally complemented by behavior guidance techniques and long-term follow-up. Given the increasing use of DGA in pediatric dentistry, further research with standardized methods and longer follow-up periods is needed to better understand its psychological effects and ensure safe and effective anxiety management in children.

## Supplementary Information

Supplementary Material 1: Supplementary Table 1. Search strategy

Supplementary Material 2: Supplementary Table 2. List of excluded articles with reasons

Supplementary Material 3: Supplementary Table 3. Risk of bias within studies. Table A. Newcastle-Ottawa assessment scale for case-control studies. Table B. Quality assessment tool for Before-Afterstudies with no control group

Supplementary Material 4: Supplementary Table 4. Certainty of evidence assessment (GRADE)

## Data Availability

The data supporting this study's findings are available from the corresponding author upon request.

## Abbreviations

DFA: Dental fear and anxiety
DGA: Dental general anesthesia
GA: General anesthesia
DBS: Dental behavior support
ECC: Early childhood caries
ASA: American Society of Anesthesiologists
CFSS-DS/CFSS-DSf: Children’s Fear Survey Schedule-Dental Subscale/(faces version)
MCDAS/MCDASf: Modified Child Dental Anxiety Scale/(faces version)
VPT: Venham Picture Test
FIS: Facial Image Scale
DFS: Dental Fear Survey
CDFP: Children’s Dental Fear Picture Test
DAQ: Dental Anxiety Question
PRO: Patient-reported outcome
NOS: Newcastle–Ottawa Scale
NIH tool: National Institutes of Health quality assessment tool
PRISMA: Preferred Reporting Items for Systematic Reviews and Meta-Analyses
GRADE: Grading of Recommendations, Assessment, Development and Evaluations
PROSPERO: International Prospective Register of Systematic Reviews
PICO: Population, Intervention, Comparison, Outcome
SMD: Standardized mean difference; CI: Confidence interval
I^2^: Higgins’ I-squared statistic
SD: Standard deviation
