# Supplementary material for: The effect of dental rehabilitation under general anesthesia on dental anxiety in children: a systematic review and meta-analysis
Source: BMC Oral Health. 2025 Dec 29;25:1953. doi: 10.1186/s12903-025-07334-y (PMC12751333; doi:10.1186/s12903-025-07334-y)
Supplement: Supplementary file 1 — Supplementary Material 1: Supplementary Table 1. Search strategy [file 12903_2025_7334_MOESM1_ESM.docx]

**Supplementary Table 1.**

**Search strategy**

| [3,202](https://www.webofscience.com/wos/woscc/summary/6b65b507-da8e-43a8-abba-8d0a10c23a5d-013d527304/relevance/1) | **"dental anxiety"** (Topic) or **"dental stress analysis"** (Topic) or **"dental fear"** (Topic) or **"dental phobia"** (Topic) or **"dental stress"** (Topic) or **"odontophobic"** (Topic) | Web of Science |
| --- | --- | --- |
| [51,329](https://www.webofscience.com/wos/woscc/summary/06e3f5bc-69db-404c-9d81-d5c916dc2d41-013d52dabb/relevance/1) | **"general anesthesia"** (Topic) or **"dental general anesthesia"** (Topic) |  |
| [80](https://www.webofscience.com/wos/woscc/summary/688d6a6c-1923-426f-85a9-07d725c65d10-013d52e420/relevance/1) | **#2 AND #1** |  |
| [97,442](https://pubmed.ncbi.nlm.nih.gov/?term=%28%28%22Anesthesia%2C+General%22%5BMesh%5D%29+OR+%28%22general+anesthesia%22%29%29+OR+%28%22dental+general+anesthesia%22%29&sort=date&size=200) | **(("Anesthesia, General"[Mesh]) OR ("general anesthesia")) OR ("dental general anesthesia")** Sort by: **Most Recent**  "anesthesia, general"[MeSH Terms] OR "general anesthesia"[All Fields] OR "dental general anesthesia"[All Fields] | Pubmed |
| [23,691](https://pubmed.ncbi.nlm.nih.gov/?term=%22Dental+Anxiety%22%5BMeSH+Terms%5D+OR+%22Dental+Stress+Analysis%22%5BMeSH+Terms%5D+OR+%22Dental+fear%22%5BText+Word%5D+OR+%22Dental+Phobia%22%5BText+Word%5D+OR+%22dental+stress%22%5BText+Word%5D+OR+%22Odontophobia%22%5BText+Word%5D+OR+%22dental+pain%22%5BText+Word%5D+OR+%22dental+Pain+Management%22%5BText+Word%5D&sort=date&size=200) | **"Dental Anxiety"[MeSH Terms] OR "Dental Stress Analysis"[MeSH Terms] OR "Dental fear"[Text Word] OR "Dental Phobia"[Text Word] OR "dental stress"[Text Word] OR "Odontophobia"[Text Word] OR "dental pain"[Text Word] OR "dental Pain Management"[Text Word]** Sort by: **Most Recent**  "Dental Anxiety"[MeSH Terms] OR "Dental Stress Analysis"[MeSH Terms] OR "Dental fear"[Text Word] OR "Dental Phobia"[Text Word] OR "dental stress"[Text Word] OR "Odontophobia"[Text Word] OR "dental pain"[Text Word] OR "dental Pain Management"[Text Word] |  |
| [245](https://pubmed.ncbi.nlm.nih.gov/?term=%28%22Dental+Anxiety%22%5BMeSH+Terms%5D+OR+%22Dental+Stress+Analysis%22%5BMeSH+Terms%5D+OR+%22Dental+fear%22%5BText+Word%5D+OR+%22Dental+Phobia%22%5BText+Word%5D+OR+%22dental+stress%22%5BText+Word%5D+OR+%22Odontophobia%22%5BText+Word%5D+OR+%22dental+pain%22%5BText+Word%5D+OR+%22dental+Pain+Management%22%5BText+Word%5D%29+AND+%28%28%28%22Anesthesia%2C+General%22%5BMesh%5D%29+OR+%28%22general+anesthesia%22%29%29+OR+%28%22dental+general+anesthesia%22%29%29&sort=date&size=200) | **("Dental Anxiety"[MeSH Terms] OR "Dental Stress Analysis"[MeSH Terms] OR "Dental fear"[Text Word] OR "Dental Phobia"[Text Word] OR "dental stress"[Text Word] OR "Odontophobia"[Text Word] OR "dental pain"[Text Word] OR "dental Pain Management"[Text Word]) AND ((("Anesthesia, General"[Mesh]) OR ("general anesthesia")) OR ("dental general anesthesia"))** Sort by: **Most Recent**  ("Dental Anxiety"[MeSH Terms] OR "Dental Stress Analysis"[MeSH Terms] OR "Dental fear"[Text Word] OR "Dental Phobia"[Text Word] OR "dental stress"[Text Word] OR "Odontophobia"[Text Word] OR "dental pain"[Text Word] OR "dental Pain Management"[Text Word]) AND ("anesthesia, general"[MeSH Terms] OR "general anesthesia"[All Fields] OR "dental general anesthesia"[All Fields]) |  |
| 22,195 | ( TITLE-ABS-KEY ( "dental anxiety" ) OR TITLE-ABS-KEY ( "dental stress analysis" ) OR TITLE-ABS-KEY ( "dental fear" ) OR TITLE-ABS-KEY ( "dental phobia" ) OR TITLE-ABS-KEY ( "dental stress" )OR TITLE-ABS-KEY ( odontophobia ) ) | Scopus |
| 135,923 | ( TITLE-ABS-KEY ( "general anesthesia" ) OR TITLE-ABS-KEY ( "dental general anesthesia" ) ) |  |
| 302 | ( ( TITLE-ABS-KEY ( "dental anxiety" ) OR TITLE-ABS-KEY ( "dental stress analysis" ) OR TITLE-ABS-KEY ( "dental fear" ) OR TITLE-ABS-KEY ( "dental phobia" ) OR TITLE-ABS-KEY ( "dental stress" ) ORTITLE-ABS-KEY ( odontophobia ) ) ) AND ( ( TITLE-ABS-KEY ( "general anesthesia" ) OR TITLE-ABS-KEY ( "dental general anesthesia" ) ) ) |  |
| 14 | dental anxiety, dental fear, dental phobia, dental stress analysis, general anesthesia, and dental general anesthesia | Google scholar |
| 641 | 15 january 2025 | overall |
