# Supplementary material for: The effect of dental rehabilitation under general anesthesia on dental anxiety in children: a systematic review and meta-analysis
Source: BMC Oral Health. 2025 Dec 29;25:1953. doi: 10.1186/s12903-025-07334-y (PMC12751333; doi:10.1186/s12903-025-07334-y)
Supplement: Supplementary file 2 — Supplementary Material 2: Supplementary Table 2. List of excluded articles with reasons [file 12903_2025_7334_MOESM2_ESM.docx]

**Supplementary Table 2**

**List of excluded articles with reasons.**

| Reason to exclude | Year | Country | Study | ID |
| --- | --- | --- | --- | --- |
| DFA measured before and during induction of GA not after. | 2010 | Jordan | Al-Jundi SH, Mahmood AJ. Factors affecting preoperative anxiety in children undergoing general anaesthesia for dental rehabilitation. Eur Arch Paediatr Dent. 2010;11(1):32-7. | 1 |
| Just preoperative percentage of Venham scale reported. | 2004 | United Kingdom | Atan S, Ashley P, Gilthorpe MS, Scheer B, Mason C, Roberts G. Morbidity following dental treatment of children under intubation general anaesthesia in a day-stay unit. Int J Paediatr Dent. 2004;14(1):9-16. | 2 |
| DFA Measurements after DGA were done by telephone interview. Missing English full text | 2004 | United Kingdom | Balmer R, O'Sullivan EA, Pollard MA, Curzon MEJ. Anxiety related to dental general anaesthesia: Changes in anxiety in children and their parents. European Journal of Paediatric Dentistry. 2004;5(1):9-14. | 3 |
| Cases were adults not children. | 1986 | Sweden | Berggren U. LONG-TERM EFFECTS OF 2 DIFFERENT TREATMENTS FOR DENTAL FEAR AND AVOIDANCE. Journal of Dental Research. 1986;65(6):874-6. | 4 |
| Just VAS median reported. | 2005 | United Kingdom | Campbell C, Hosey MT, McHugh S. Facilitating coping behavior in children prior to dental general anesthesia: A randomized controlled trial. Paediatric Anaesthesia. 2005;15(10):831-8. | 5 |
| Measurements were just prior to GA. | 2001 | United Kingdom | Carson P, Freeman R. Dental caries, age and anxiety: factors influencing sedation choice for children attending for emergency dental care. Community Dent Oral Epidemiol. 2001;29(1):30-6. | 6 |
| Missing English full text. | 2007 | United Kingdom | Gazal G, Mackie IC. Distress related to dental extraction for children under general anaesthesia and their parents. Eur J Paediatr Dent. 2007;8(1):7-12. | 7 |
| DAS median reported. | 1993 | Sweden | Hakeberg M, Berggren U, Carlsson SG, Gröndahl HG. Long-term effects on dental care behavior and dental health after treatments for dental fear. Anesth Prog. 1993;40(3):72-7. | 8 |
| Cases were adults not children. | 2017 | United Kingdom | Haworth S, Dudding T, Waylen A, Thomas SJ, Timpson NJ. Ten years on: Is dental general anaesthesia in childhood a risk factor for caries and anxiety? Br Dent J. 2017;222(4):299-304. | 9 |
| DFA measurements were just prior to GA. | 2006 | United Kingdom | Hosey MT, Macpherson LMD, Adair P, Tochel C, Burnside G, Pine C. Dental anxiety, distress at induction and postoperative morbidity in children undergoing tooth extraction using general anaesthesia. British Dental Journal. 2006;200(1):39-43. | 10 |
| DFA measurements were just prior to GA. | 2022 | United Kingdom | Hua L, Busuttil-Naudi A, Keightley AJ. Do paediatric patient-related factors affect the need for a dental general anaesthetic? British Dental Journal. 2022;233(5):407-12. | 11 |
| Missing English full text. | 2014 | Iran | Jabarifar SE, Ahmadi Rozbahani N, Javadi nejad S, Hosseini L. A Comparative Evaluation of Fear and Anxiety in (3-6) Year Old Children Following Treatment under General Anesthesia and Outpatient Dental Treatment. Journal of Mashhad Dental School. 2014;38(1):9-16. | 12 |
| Data were reported as percentage. | 1998 | Israel | Kupietzky A, Blumenstyk A. Comparing the behavior of children treated using general anesthesia with those treated using conscious sedation. ASDC J Dent Child. 1998;65(2):122-7. | 13 |
| Before-after DGA CFSS-DS mean without SD reported. | 2024 | china | Li M, Xiong H, Li M, Chee WW, Chen K. Change in oral health-related behaviours of children before and after dental treatments under general anaesthesia. J Clin Pediatr Dent. 2024;48(3):94-100. | 14 |
| Not valid questionnaires to assess dental anxiety. | 2017 | United Kingdom | Ramdaw A, Hosey MT, Bernabé E. Factors associated with use of general anaesthesia for dental procedures among British children. British Dental Journal. 2017;223(5):339-45. | 15 |
| DFA measurements were just prior to GA. | 2014 | Finland | Taskinen H, Kankaala T, Rajavaara P, Pesonen P, Laitala ML, Anttonen V. Self-reported causes for referral to dental treatment under general anaesthesia (DGA): a cross-sectional survey. Eur Arch Paediatr Dent. 2014;15(2):105-12. | 16 |
| Cases were adults not children. | 2011 | Germany | Wannemueller A, Joehren P, Haug S, Hatting M, Elsesser K, Sartory G. A practice-based comparison of brief cognitive behavioural treatment, two kinds of hypnosis and general anaesthesia in dental phobia. Psychother Psychosom. 2011;80(3):159-65. | 17 |
| Missing English full text. | 2015 | china | Zhang HM, Xia B, Wang JH, Chen XX, Ge LH. [Influence of the effect of general anaesthesia and restraint during dental treatment on dental anxiety and behavior in children]. Beijing Da Xue Xue Bao Yi Xue Ban. 2015;47(1):134-9. | 18 |
