# Supplementary material for: The effect of dental rehabilitation under general anesthesia on dental anxiety in children: a systematic review and meta-analysis
Source: BMC Oral Health. 2025 Dec 29;25:1953. doi: 10.1186/s12903-025-07334-y (PMC12751333; doi:10.1186/s12903-025-07334-y)
Supplement: Supplementary file 3 — Supplementary Material 3: Supplementary Table 3. Risk of bias within studies. Table A. Newcastle-Ottawa assessment scale for case-control studies. Table B. Quality assessment tool for Before-Afterstudies with no control group [file 12903_2025_7334_MOESM3_ESM.docx]

**Supplementary Table 3. Risk of bias within studies.**

| Study | **Selection** | | | | **Comparability** | **Exposure** | | | **Total** | **Quality rating** |
| --- | --- | --- | --- | --- | --- | --- | --- | --- | --- | --- |
|  | Definition of case | Represent-ativeness of cases | Selection of controls | Definition of controls |  | Assessment of exposure | Same methods of ascertainment for cases and controls | Non response rate |  |  |
| Zhou, F., et al. (2022) | + | + | - | + | + | - | - | - | 4 | medium |
| Aldossari, G. S., et al. (2019) | + | + | - | + | + | - | - | + | 5 | medium |
| Klaassen, M. A., et al. (2009) | + | + | - | + | + | - | + | - | 5 | medium |

Table A. Newcastle-Ottawa assessment scale for case-control studies.

**Supplementary Table 3. Risk of bias within studies.**

| **Author** | **Q1** | **Q2** | **Q3** | **Q4** | **Q5** | **Q6** | **Q7** | **Q8** | **Q9** | **Q10** | **Q11** | **Q12** | **Total score** | **Quality rating** |
| --- | --- | --- | --- | --- | --- | --- | --- | --- | --- | --- | --- | --- | --- | --- |
| Mathew, M. G., et al. (2023) | Y | Y | Y | Y | Y | Y | Y | NR | Y | Y | N | NA | 9 (11) | Good |
| Heaton, L. J., et al. (2023) | Y | Y | Y | N | Y | Y | Y | NR | Y | Y | Y | NA | 9 (11) | Good |
| Duruk, G., et al. (2021). | Y | Y | Y | Y | Y | Y | Y | NR | Y | Y | N | NA | 9 (11) | Good |
| Guney, S. E., et al. (2018) | Y | Y | Y | Y | NR | Y | Y | NR | Y | Y | N | NA | 8 (11) | Fair |
| Cantekin, K., et al. (2014) | Y | Y | Y | Y | Y | Y | Y | NR | Y | Y | N | NA | 9 (11) | Good |
| Klaassen, M. A., et al. (2009) | Y | Y | Y | N | Y | Y | Y | NR | Y | Y | N | NA | 8 (11) | Fair |
| Klaassen, M. A., et al. (2008) | Y | Y | Y | N | N | Y | Y | NR | N | Y | N | NA | 6 (11) | Fair |

Table B. Quality assessment tool for Before-After (Pre-Post) studies with no control group.
