# Supplementary material for: The effect of dental rehabilitation under general anesthesia on dental anxiety in children: a systematic review and meta-analysis
Source: BMC Oral Health. 2025 Dec 29;25:1953. doi: 10.1186/s12903-025-07334-y (PMC12751333; doi:10.1186/s12903-025-07334-y)
Supplement: Supplementary file 4 — Supplementary Material 4: Supplementary Table 4. Certainty of evidence assessment (GRADE) [file 12903_2025_7334_MOESM4_ESM.pdf]

Author(s): Elham Keykha, Samira Hajisadeghi, Mohammad Taha Heidari
Question: Post-DGA/DGA-cases DFA score compared to Baseline (pre-DGA)/Control DFA score in Children who undergone DGA
Setting: Healthy children who have undergone DGA.
Bibliography:

Certainty assessment							№ of patients		Effect		Certainty	Importance
№ of studies	Study design	Risk of bias	Inconsistency	Indirectness	Imprecision	Other considerations	Post-DGA/DGA-cases DFA score	Baseline (pre-DGA)/Control DFA score	Relative (95% CI)	Absolute (95% CI)		
**New outcome (follow-up: range 2 weeks to 16 weeks)**												
7	non-randomised studies	serious[a]	very serious[b]	not serious	serious[c]	none	673	673	-	SMD **0 SD** (1.993 lower to 0.593 higher)	⨁◯◯◯ Very low[a,b,c]	CRITICAL
**New outcome (follow-up: range 1 months to 2 years)**												
3	non-randomised studies	not serious	serious[d]	not serious	serious[c]	none	77	107	-	SMD **0 SD** (1.048 lower to 1.313 higher)	⨁◯◯◯ Very low[c,d]	CRITICAL
**New outcome (follow-up: range 1 years to 3 years)**												
2	non-randomised studies	not serious	serious[e]	not serious	serious[c]	none	63	75	-	SMD **0 SD** (0.152 lower to 1.853 higher)	⨁◯◯◯ Very low[c,e]	CRITICAL

CI: confidence interval; **SMD:** standardised mean difference

Explanations

a. A substantial proportion of the evidence comes from before–and–after studies without external controls. DFA outcomes were self/parent-reported or assessed by practitioners without blinding, and the timing of assessment varied, introducing potential performance and detection bias.
b. Very high statistical heterogeneity (I^2 = 98.8%), mixed directions of effect across studies, and substantial clinical/measurement variability (different follow-up windows and treatment burdens) not resolved by sensitivity or subgroup analyses.
c. The 95% CI is very wide and crosses the minimally important difference (MID) threshold of ±0.20. It extends well below −0.20, suggesting appreciable benefit, and above +0.20, suggesting possible harm or no effect.
d. Very high heterogeneity (I^2=92.6%); mixed directions; different follow-up windows.
e. Very high heterogeneity (I^2=85.5%); mixed directions; different follow-up windows.
